# Gleanings

**Published:** 1876-05

**Authors:** 


### GLEANINGS.

Dr. Richardson reports to the Obstetrical Society of Boston two cases of *sub-acute cystitis* following parturition in primiparæ. The symptoms were tenderness of hypogaster, dysuria, elevated temperature and pulse, with general febrile disturbance; the milk and lochia were normal; the urine loaded with mucus and more or less pus. The treatment consisted of fomentations, small doses of morphia, diuretics and washing out the bladder with, first warm water, and afterwards water medicated with three to six drops of carbolic acid to the pint. These injections were made through the double catheter, and were followed always by marked relief to all the symptoms. A prompt recovery ensued in all the cases. Prolonged pressure upon the bladder in primapara labor is assigned as the cause of the cystitis.

Sir Henry Thompson, a most eminent authority upon the subject of urethral surgery, in a recent lecture deprecates the tendency of the times to multiply supposed strictures of the urethra, and to subject that delicate tube to harsh and unnecessary dilatations. "When, therefore, we are consulted by a patient for certain troubles for which it is important to know whether urethral obstructions be a cause or not, we should not adopt the unnecessary course of introducing very large instruments, but should take a flexible bougie, well curved toward the point, with a blunt end, not larger as a rule than No. 10 or 11 of the English scale, and pass it very gently and slowly into the bladder. If it goes easily, above all, if it is withdrawn without being held, and slides out with perfect facility, the patient has no stricture, and, as far as obstruction is concerned, needs no use of instruments."

Prof. Bischoff, of Munich, the veteran searcher into the mysteries of generation, says, in a recent paper upon the physiology of menstruation and of procreation: "Although cases of early conception, or conception without preceding menstrual hemorrhage, or of extra uterine pregnancy, prove the independence of ovulation and conception from the changes and hemorrhage in the uterus, in those cases they prove it only as a *rare exception*, not as a rule, and by no means refute the law that ovulation, formation of a decidua, and uterine hemorrhage are necessary to the normal development of the whole process of procreation.

"The occurrence of regular uterine hemorrhage independently of the ovaries (when they are degenerated or have been removed) is possible, and explicable *on the ground of a habit* acquired after many years of regular typical congestion and hemorrhage coincident with ovulation.

"The order in which the stages of the process of generation follow each other are: 1, ovulation in the ovaries; 2, formation of decidua and hemorrhage in the uterus; and, 3, action of the sexual impulse to bring the two generative substances together.

"It is an indispensable law for the human race, as well as for animal creation, that *menstruation depends on the maturation and discharge of an ovum from the ovary,* and that impregnation and conception directly depend on this process. The ripening and discharge of an ovum no more depend on sexual intercourse than does menstruation."

